# Leveraging logistic models to enhance nutrient dynamics modeling in intercropped spring wheat with varied nitrogen and phosphorus fertilization strategies

**DOI:** 10.1371/journal.pone.0314264

**Published:** 2024-12-05

**Authors:** Yue Feng, Ying Ma, Renqiang Deng, Bo Wang, Haiyan Tian, Xin Liu, Qi Dong, Aiping Wang, Sumera Anwar

**Affiliations:** 1 College of Agriculture, Shanxi Agriculture University, Shanxi, Taigu, China; 2 Department of Botany, Government College Women University Faisalabad, Faisalabad, Pakistan; Universidade Federal de Minas Gerais, BRAZIL

## Abstract

Intercropping systems offer substantial benefits in crop yield nd nutrient absorption. Utilizing logistic models, we simulated the dynamic of nutrient uptake and accumulation in spring wheat and the impact of different planting patterns and compound fertilizer application rates on spring wheat yield. We conducted a field experiment involving two planting patterns: spring wheat monoculture (MS) and spring wheat-pea intercropping (MI), with five compound fertilizer applications: C0 (0 kg ha^-1^), C1 (480 kg ha^-1^), C2 (540 kg ha^-1^), C3 (600 kg ha^-1^), and C4 (660 kg ha^-1^). We assessed spring wheat yield and aboveground nitrogen (N) and phosphorus (P) accumulation under different planting patterns and fertilization treatments. Results revealed that intercropping significantly increased spike number, grains per spike, and grain yield of spring wheat by 3.7%, 6.3%, and 13.3%, respectively, compared to monoculture. Fertilization treatments notably enhanced average spring wheat grain yield, with C2 performing optimally. Logistic model analysis indicated that under intercropping, the maximum accumulated aboveground N and N uptake rate (v) of spring wheat was 11.4% and 13.2% higher, and the maximum accumulated P and maximum P uptake rate (V_max_) were 11.3% and 9.5% higher, respectively, compared to monoculture. Intercropped spring wheat under C2 exhibited the highest P accumulation among all treatments. In conclusion, both intercropping and fertilization can enhance N and P uptake and accumulation in spring wheat, thereby boosting yield. Optimized yield can be achieved under C2 (540 kg h^-1^) with a 10% reduction in fertilizer application. Thus effective control of fertilizer application is pivotal for maximizing the yield advantage of the spring wheat/pea intercropping system.

## 1. Introduction

Intercropping refers to the farming technique of growing two or more crops simultaneously on the same land, with different planting and harvesting times that allow for partial overlap in growth periods [1]. This planting strategy optimizes the utilization of agricultural resources such as land, nutrients, water, heat, and radiation over both time and space [2].

In China, intercropping cereals with legumes is a traditional agricultural practice, especially prevalent in regions with relatively low soil fertility [3]. This cropping system not only boosts and stabilizes yields but also plays a vital role in ensuring food security [4].

Nitrogen and phosphorus are two essential macronutrients critical for plant growth and development [5, 6]. Nitrogen fertilizers enhance wheat nitrogen uptake [7], promote wheat growth, significantly improve wheat quality [8], and increase winter wheat grain yield [9]. However, excessive nitrogen application over time can reduce available soil phosphorus [10] underscoring the importance of balanced nitrogen and phosphorus fertilizer inputs to optimize crop nutrient uptake and utilization.

Research indicates that combined nitrogen and phosphorus fertilizer application significantly influences wheat yield and quality [11–13]. This practice can mitigate interspecific competition between maize and soybean, enhance the competitiveness and nutritional competition ratio of relay intercropping systems, and facilitate nitrogen and phosphorus uptake [14].

Wheat intercropping with legumes has been widely used particularly under low soil N. For instance, spring wheat intercropped with alfalfa improved the soil structure and mitigated soil salinity through ion balance [15]. Similarly, spring wheat intercropped with legumes such as pea, bean, lupin, and vetch reduced weed growth and enhanced grain protein content [16]. Nevertheless, the success of intercropping hinges on various factors including intercrop selection and nutrient management [17].

A balanced application of nitrogen, phosphorus, and potassium fertilizers is crucial for promoting crop nutrient uptake and utilization [18]. Thus, investigating nutrient uptake and utilization under varying fertilizer combinations is imperative for elucidating the competitive and inhibitory interactions between nutrients. The logistic model is a widely used method for predicting population growth [19], crop growth and development [20], and nutrient accumulation. This model depicts the complex dynamic changes in population growth, plant height, etc, and can effectively simulate key parameters of nitrogen uptake in wheat and chickpea intercropping, elucidating dynamic patterns of nitrogen uptake and accumulation under varying nitrogen levels [21].

While numerous studies have explored nutrient uptake advantages in intercropping systems, few have delved into the dynamics and processes governing nutrient uptake, distribution, utilization, and interactions within intercropping systems. This experiment aims to study the uptake patterns of nitrogen and phosphorus at different growth stages of spring wheat under wheat-pea intercropping conditions through logistic model simulation and analysis. By examining the relationships between yield and nitrogen and phosphorus accumulation in spring wheat in response to different planting patterns and compound fertilizer application rates, while maintaining fixed nitrogen, phosphorus, and potassium nutrient ratio. Our study may provide a theoretical basis for rational fertilization of cereal/legume intercropping systems.

## 2 Materials and methods

### 2.1 Experimental time, location, and materials

The experiment took place on a farm at Shanxi Agricultural University (Taigu District, Jinzhong City, Shanxi Province) from March to June 2022. The geographical coordinates of the experiment site are 112°34’ E, 37°25′ N. The climate in this region is characterized as warm temperate continental, with an average annual temperature ranging from 5–10°C and an annual rainfall of 458 mm. The soil type is loam, with 21.96 g kg^-1^ organic matter, 0.94 g kg^-1^ total nitrogen, 36.65 mg kg^-1^ alkali-hydrolyzable nitrogen, 0.96 g kg^-1^ total phosphorus, and 18.51 mg kg^-1^ available phosphorus. The spring wheat variety used was Ningchun 4, planted on March 16 and harvested on June 28. The pea variety was Zhongwan 11, planted on March 16 and harvested on June 15. The compound fertilizer applied was Ezhong compound fertilizer with total nutrients ≥51% and a ratio of N∶P_2_O_5_∶K_2_O = 9∶13∶9. The temperature and precipitation in the field test sites during the whole growing period from 2022 to 2023 is presented in S1 Fig.

### 2.2 Experimental design

The experiment comprised 2 planting patterns: spring wheat monoculture (MS) and spring wheat-pea intercropping (MI) along with five fertilization treatments: C0 (0 kg ha^-1^, no fertilization), C1 (480 kg ha^-1^, 20% reduction), C2 (540 kg ha^-1^, 10% reduction), C3 (600 kg ha^-1^, conventional amount), and C4 (660 kg ha^-1^, 10% increase). There were 3 replications for each treatment resulting in a total of 30 plots with each plot measuring 7.5 m^2^ (3 m×2.5 m). The experimental layout followed a randomized complete block design.

Spring wheat was planted in rows using strip sowing, and peas were planted in rows using hole sowing with 15 holes per row and 2 seeds per hole. Each plot was planted with 12 rows at a row spacing of 12 cm. Intercropped plots had 2 rows of spring wheat alternating with 2 rows of peas with a total of six rows of spring wheat and six rows of peas in each plot. The four rows of wheat were used for nutrient analysis and two rows for yield measurement.

The spring wheat sowing rate was 225 kg h^-1^, and the pea plant density was 480,000 plants kg h^-1^. Compound fertilizer was applied as base fertilizer before sowing in a one-time application. No topdressing was applied during the whole growth period, and other management practices were the same as local practices.

### 2.3 Measurements and methods

#### 2.3.1 Sampling times and methods

Three representative spring wheat plants were randomly collected from each plot at the tillering, jointing, heading, milky stage, and maturity. Plants were dried at 110°C for 30 min, then oven dried at 80°C for 24 h until constant weight. Samples were weighed, chopped, ground into powder using a pulverizer, and stored in sealed bags for plant nutrient analysis.

#### 2.3.2 Yield

Mature spring wheat from the 2 rows of yield measurement area was collected to determine the total spike number, grains per spike, and 1000-grain weight. Yield was calculated based on area using the formula:

Yield (kg h^**-1**^) = spikes per hectare (10^**4**^ h^**-1**^) × grains per spike × 1000-grain weight (g)

#### 2.3.3 Plant nutrient content

Spring wheat plant N and P content was determined using H_2_SO_4_-H_2_O_2_ digestion and measured by spectrophotometry. The aboveground N and P contents were determined at five growth stages from tillering to maturity. The total N and P accumulation was calculated by multiplying the aboveground dry biomass with N and P content. The N and P uptake at a particular growth stage was measured from the difference in N and P accumulation from the previous stage.

### 2.4 Nutrient use efficiency and yield increase rate calculations

Fertilizer partial factor productivity (kg kg^-1^) = Grain yield / Fertilizer application rate

Agronomic efficiency (kg kg^-1^) = (Grain yield with fertilization—Grain yield without fertilization) / Fertilizer application rate

Yield increase rate (%) = (Grain yield with fertilization–Grain yield without fertilization) / Grain yield without fertilization × 100%

### 2.5 Simulation of spring wheat aboveground nitrogen and phosphorus accumulation dynamics

Nitrogen and phosphorus accumulation dynamics of spring wheat were fitted using the logistic growth mode [7]. The OriginPro 2022 software Slogistic1 simulation was used to generate aboveground nitrogen and phosphorus accumulation curves and key parameters. Differentiating the curves gave nitrogen and phosphorus uptake rate curves, with the following model [22]:

$$y_{t}=\frac{N\;or\;P}{1+exp[v(T-t)]}$$

$$v_{t}=v\times y_{t}(1‐y_{t}/N)$$

$$V_{max}=N\;or\;P\times v/4$$

where y_t_ is the accumulated aboveground nitrogen or phosphorus content on day t of the spring wheat growth period (kg h^-1^); N and P are the maximum accumulated aboveground nitrogen and phosphorus content (kg h^-1^); v is the spring wheat initial nitrogen or phosphorus uptake rate, (kg h^-1^ d^-1^); T is the time for spring wheat to reach maximum nitrogen or phosphorus uptake rate (d); t is spring wheat growth time (d); v_t_ is the nitrogen or phosphorus uptake rate on day t of the spring wheat growth period (kg h^-1^ d^-1^); V_max_ is the maximum spring wheat nitrogen or phosphorus uptake rate (kg h^-1^ d^-1^).

### 2.6 Statistical analysis

Microsoft Office Excel 2019 was used for data processing, SPSS for analysis of variance and multiple comparisons, and OriginPro 2022 for plotting.

## 3 Results

### 3.1 Yield and yield components

The effect of different fertilizer rates was measured on the yield of spring wheat grown under monocropping and intercropping with pea plants (Table 1). The fertilizer rate had significantly affected all yield traits. The planting pattern had a significant effect on spike number and grain yield. Grain number per spike, grain weight, and grain yield were significantly affected by the interactive effect of cropping and fertilizer with a maximum value at C2 (600 kg ha^-1^) under intercropping and a minimum under monocropping without fertilizer.

**Table 1:** Spring wheat yield and yield components.

Treatments			Number of spikes (10^4^ plants ha^-1^)	Grain number per spike (spike)	Thousand grains weight (g)	Grain yield (kg ha^-1^)
Planting method		MS	304.75±5.70 b	59.77±0.55 b	26.88±0.08 a	4923.30±23.79 b
		MI	316.05±6.42 a	63.55±0.88 a	27.68±0.33 a	5579.28±86.58 a
Fertilizers		C0	287.47±3.34 e	55.33±0.49 d	26.31±0.19 b	4185.77±18.96 e
		C1	300.27±6.42 d	60.06±0.59 c	26.77±0.19 ab	4839.50±45.56 d
		C2	320.53±5.78 b	66.62±1.28 a	29.11±0.12 a	6216.31±43.65 a
		C3	312.53±10.50 c	63.89±0.37 ab	27.36±0.31 ab	5462.16±121.56 c
		C4	331.20±4.29 a	62.42±0.86 bc	26.86±0.21 ab	5552.72±46.20 b
Interaction						
MS		C0	284.80±3.70 e	53.50±0.87 d	25.93±0.02 e	3948.72±10.69 f
		C1	295.47±9.10 cde	56.13±0.82 cd	26.18±0.12 de	4341.09±17.87 e
		C2	314.67±3.50 bc	64.19±0.60 b	28.88±0.10 a	5832.47±29.62 bc
		C3	313.60±10.62 bc	62.88±0.30 b	26.82±0.08 cd	5288.61±30.65 d
		C4	315.20±1.60 bc	62.16±0.17 b	26.57±0.08 cde	5205.61±30.12 d
MI		C0	290.13±2.97 de	57.15±0.10 c	26.68±0.36 cde	4422.82±27.23 e
		C1	305.07±3.73 bcde	63.99±0.35 b	27.35±0.26 bc	5337.90±73.24 d
		C2	326.40±8.05 b	69.05±1.96 a	29.33±0.14 a	6600.15±57.68 a
		C3	311.47±10.38 bcd	64.89±0.44 b	27.89±0.54 b	5635.71±212.46 c
		C4	347.20±6.97 a	62.67±1.54 b	27.14±0.34 bc	5899.83±62.28 b
p-value	Fertilizer		**	**	**	**
	Planting method		**	ns	ns	*
	Interaction		ns	**	**	**

MS: spring wheat sole cropping, MI: spring wheat and pea intercropping: C0, C1, C2, C3 and C4 represent 480, 540, 600, and 660 kg ha^-1^ fertilization rate, respectively. Different lowercase letters in the same column indicate significant differences between treatments; * and ** indicate significance at p < 0.05 and p<0.01, respectively; ns: not significant.

Grains number per spike, 1000-grain weight, and grain yield first increased then decreased with increasing fertilization rate reaching a maximum value at C2. The number of spikes showed an increasing trend with increasing the fertilizer rate with a maximum at C4 (660 kg ha^-1^).

Grains per spike were significantly higher under C2 than those under C0, C1, and C4. 1000-grain weight reached a maximum at C2 and was significantly higher compared with that under C0. Grain yield was highest under C2 and significantly higher than all other fertilization treatments. Regardless of fertilization treatment, intercropped spring wheat had significantly higher spike number, grains per spike, and grain yield as compared to monocropped spring wheat, with an increase of 3.7%, 6.3%, and 13.3%, respectively. Fertilized spring wheat had significantly higher spike number, grains per spike, and grain yield compared to unfertilized.

### 3.2 Fertilizer utilization efficiency and yield increase rate

The fertilization treatment, planting pattern, and their interaction had significant effects on spring wheat partial factor productivity and yield increase rate (Table 2). Compared to monoculture, intercropping significantly increased compound fertilizer partial factor productivity, agronomic efficiency, and spring wheat yield increase rate by 3.9%, 67.8%, and 76.4%, respectively. All these traits were highest under C3. Overall, partial productivity was highest at C2 under intercropping and lowest at C4 under monocropping. Agronomic efficiency and yield increase rate were highest at C2 under intercropping and lowest at C1 under monocropping. The above results indicate that intercropping improved compound fertilizer partial factor productivity, agronomic efficiency, and spring wheat yield increase rate, while these traits were highest at the C2 fertilizer rate.

**Table 2:** Fertilizer utilization rate and yield increase rate of spring wheat.

Treatments			Partial productivity (kg kg^-1^)	Agronomic efficiency (kg kg^-1^)	Yield increase rate (%)
Planting method		MS	9.59±0.05 b	1.74±0.05 b	22.99±0.79 b
		MI	9.96±0.18 a	2.92±0.17 a	40.54±2.13 a
Fertilizers		C1	9.92±0.10 b	2.15±0.09 b	28.82±1.02 b
		C2	8.35±0.08 d	2.07±0.07 b	32.88±0.88 ab
		C3	11.67±0.20 a	2.97±0.19 a	34.96±2.64 a
		C4	9.17±0.07 c	2.13±0.08 b	30.41±1.31 b
Interaction					
MS		C1	9.04±0.04 cd	0.82±0.03 d	9.94±0.30 e
		C2	10.80±0.05 b	3.49±0.07 b	47.71±1.14 a
		C3	8.81±0.05 d	2.23±0.05 c	33.93±0.77 b
		C4	7.89±0.05 e	1.90±0.05 c	31.83±0.95 bc
MI		C1	11.12±0.15 b	1.91±0.16 c	20.70±1.74 d
		C2	12.22±0.11 a	4.03±0.06 a	49.23±0.62 a
		C3	9.39±0.35 c	2.02±0.34 c	27.41±4.51 c
		C4	8.94±0.09 cd	2.24±0.10 c	33.41±1.66 bc
p-value	Fertilizer		**	**	*
	Planting method		**	**	**
	Interaction		**	**	**

MS: spring wheat sole cropping, MI: spring wheat and pea intercropping: C0, C1, C2, C3 and C4 represent 480, 540, 600, and 660 kg ha^-1^ fertilization rate, respectively. Different lowercase letters in the same column indicate significant differences between treatments

* and ** indicate significance at p < 0.05 and p<0.01, respectively; ns: not significant.

### 3.3 Nitrogen uptake by aboveground parts of spring wheat

The aboveground N accumulation of spring wheat was gradually increased with the plant growth stage (Table 3). Intercropping significantly increased aboveground N accumulation at the tillering, jointing, heading, and maturity stages by 29%, 35%, 23%, and 16%, respectively, compared to monoculture.

**Table 3:** Cumulative nitrogen uptake and stage uptake in spring wheat.

Growth stage	Fertilization treatment	Total N accumulation (kg ha^-1^)		N uptake at growth stage (kg ha^-1^)	

		MS	MI	MS	MI
Tillering	C0	23.22 h	31.42 g	23.22 h	31.42 g
	C1	43.97 f	56.26 c	43.97 f	56.26 c
	C2	48.93 e	73.93 b	48.93 e	73.93 b
	C3	52.30 d	52.77 d	52.30 d	52.77 d
	C4	58.30 c	77.06 a	58.30 c	77.06 a
	Average	45.34 B	58.29 A	45.34 B	58.29 A
Jointing	C0	39.77 f	65.12 e	16.55 h	33.70 f
	C1	74.75 de	92.89 cd	30.78 g	36.63 f
	C2	117.25 b	145.40 a	68.32 c	71.47 b
	C3	98.92 bc	150.49 a	46.62 d	97.72 a
	C4	93.51 cd	117.69 b	35.22 f	40.64 e
	Average	84.84 B	114.32 A	39.50 B	56.03 A
Heading	C0	82.65 e	110.08 d	42.92 e	44.96 e
	C1	117.64 d	149.70 c	43.22 e	56.82 c
	C2	170.05 bc	201.94 a	52.81 d	56.84 c
	C3	176.43 b	189.19 ab	77.51 b	38.69 f
	C4	150.76 c	203.37 a	57.24 c	85.68 a
	Average	139.50 B	170.85 A	54.74 A	56.54 A
Filling	C0	97.02 e	125.63 de	14.34 f	15.56 ef
	C1	143.07 cd	166.95 bcd	25.09 c	17.24 e
	C2	205.05 ab	226.24 a	35.00 a	24.30 cd
	C3	199.65 ab	211.17 ab	23.22 cd	21.98 d
	C4	178.83 bc	239.07 a	28.08 b	35.71 a
	Average	164.72 A	193.81 A	25.15 A	22.96 A
Maturity	C0	99.15 h	129.70 g	2.13 g	4.07 g
	C1	163.72 f	175.45 ef	20.65 cd	8.50 f
	C2	233.29 bc	245.59 ab	28.24 b	19.35 d
	C3	208.75 cd	227.41 bcd	9.09 f	16.24 e
	C4	201.55 de	270.83 a	22.72 c	31.76 a
	Average	181.29 B	209.80 A	16.57 A	15.98 A

MS: spring wheat sole cropping, MI: spring wheat and pea intercropping: C0, C1, C2, C3 and C4 represent 480, 540, 600, and 660 kg ha^-1^ fertilization rate, respectively. Different lowercase letters in the same column indicate significant differences between treatments.

There was significantly higher N uptake at the jointing stage under the intercropping system as compared to monocropping, but no significant effect was observed from heading to the maturity stages. Among growth stages, nitrogen uptake was highest at the heading, indicating the most active growth stage for N absorption and assimilation.

The N accumulation and N uptake showed an increasing trend by increasing the fertilizer rates. The highest N accumulation and N uptake were highest under intercropping at C4 at all growth stages except at the jointing stage as indicated by the interactive effect of the cropping system and fertilizer rates. At the jointing stage, the N accumulation and uptake were first increased reaching a maximum at C3 and then decreased at C4.

### 3.4 Phosphorus uptake by aboveground parts of spring wheat

The aboveground accumulated phosphorus content of spring wheat was increased with the growth stage with an overall maximum accumulated P content of 33.97 kg ha^-1^ observed at maturity (Table 4). The planting pattern had a significant effect on aboveground phosphorus accumulation at the tillering and jointing stages while the difference in cropping system was non-significant from heading to maturity. At the tillering and jointing stages, the average accumulated phosphorus uptake under intercropping was 7.1% and 9.7% higher than that under monoculture.

**Table 4:** Cumulative phosphorus uptake and stage uptake in spring wheat.

Growth stage	Fertilization treatment	Total P accumulation (kg ha^-1^)		P uptake at growth stage (kg ha^-1^)	
		MS	MI	MS	MI
Tillering stage	C0	3.85 e	4.33 e	3.85 e	4.33 e
	C1	5.68 d	5.80 cd	5.68 d	5.80 cd
	C2	6.94 b	8.82 a	6.94 b	8.82 a
	C3	5.28 d	6.13 bcd	5.28 d	6.13 bcd
	C4	5.73 cd	6.69 bc	5.73 cd	6.69 bc
	Average	5.50 B	5.89 A	5.50 B	5.89 A
Jointing stage	C0	3.96 f	4.89 ef	0.11 d	0.56 b
	C1	6.19 cd	6.35 cd	0.51 b	0.55 b
	C2	7.49 b	9.29 a	0.55 b	0.47 b
	C3	5.50 de	7.42 b	0.22 c	1.30 a
	C4	6.19 cd	6.90 bc	0.47 b	0.20 c
	Average	5.87 B	6.37 A	0.37 B	0.48 A
Heading stage	C0	10.09 f	10.80 f	6.13 d	5.91 d
	C1	13.73 de	13.62 de	7.54 d	7.26 d
	C2	16.67 c	19.80 a	9.18 c	10.51 bc
	C3	14.75 d	18.50 b	9.24 c	11.07 b
	C4	12.88 e	19.97 a	6.69 d	13.08 a
	Average	13.62 A	14.95 A	7.76 B	8.58 A
Filling stage	C0	11.26 f	13.57 ef	1.16 f	2.78 e
	C1	16.58 def	18.30 cde	2.85 e	4.69 c
	C2	25.99 ab	29.01 a	9.35 a	9.21 a
	C3	22.59 bcd	22.29 bcd	7.85 b	3.79 d
	C4	17.92 cde	23.78 abc	5.04 c	3.80 d
	Average	18.87 A	20.02 A	5.25 A	5.07 A
Maturation stage	C0	12.21 f	13.58 f	0.96 e	0.10 f
	C1	18.27 e	23.40 d	1.68 d	5.10 a
	C2	30.83 b	33.97 a	4.84 a	4.96 a
	C3	23.74 d	25.95 cd	1.15 de	3.66 b
	C4	18.97 e	26.85 c	1.05 e	3.07 c
	Average	20.80 A	22.60 A	1.93 B	2.58 A

MS: spring wheat sole cropping, MI: spring wheat and pea intercropping: C0, C1, C2, C3 and C4 represent 480, 540, 600, and 660 kg ha^-1^ fertilization rate, respectively. Different lowercase letters in the same column indicate significant differences between treatments.

The P uptake at the tillering, jointing, heading, and maturity stages was significantly higher compared to monocrop. The interactive effect of fertilizer rate and cropping method showed that the P accumulation and P uptake were increased with increasing fertilizer rate from C0 to C2. However, with a further increase in the fertilizer rate, the phosphorus accumulation and P uptake started decreasing, except at the heading stage at which C4 showed maximum P uptake and P accumulation was significantly similar to C2. These results indicate that the aboveground P uptake was highest at the heading stage at which 13.1 kg ha^-1^ P was taken up by spring wheat.

### 3.5 Nitrogen accumulation dynamics in aboveground parts of spring wheat

**Fig 1:**
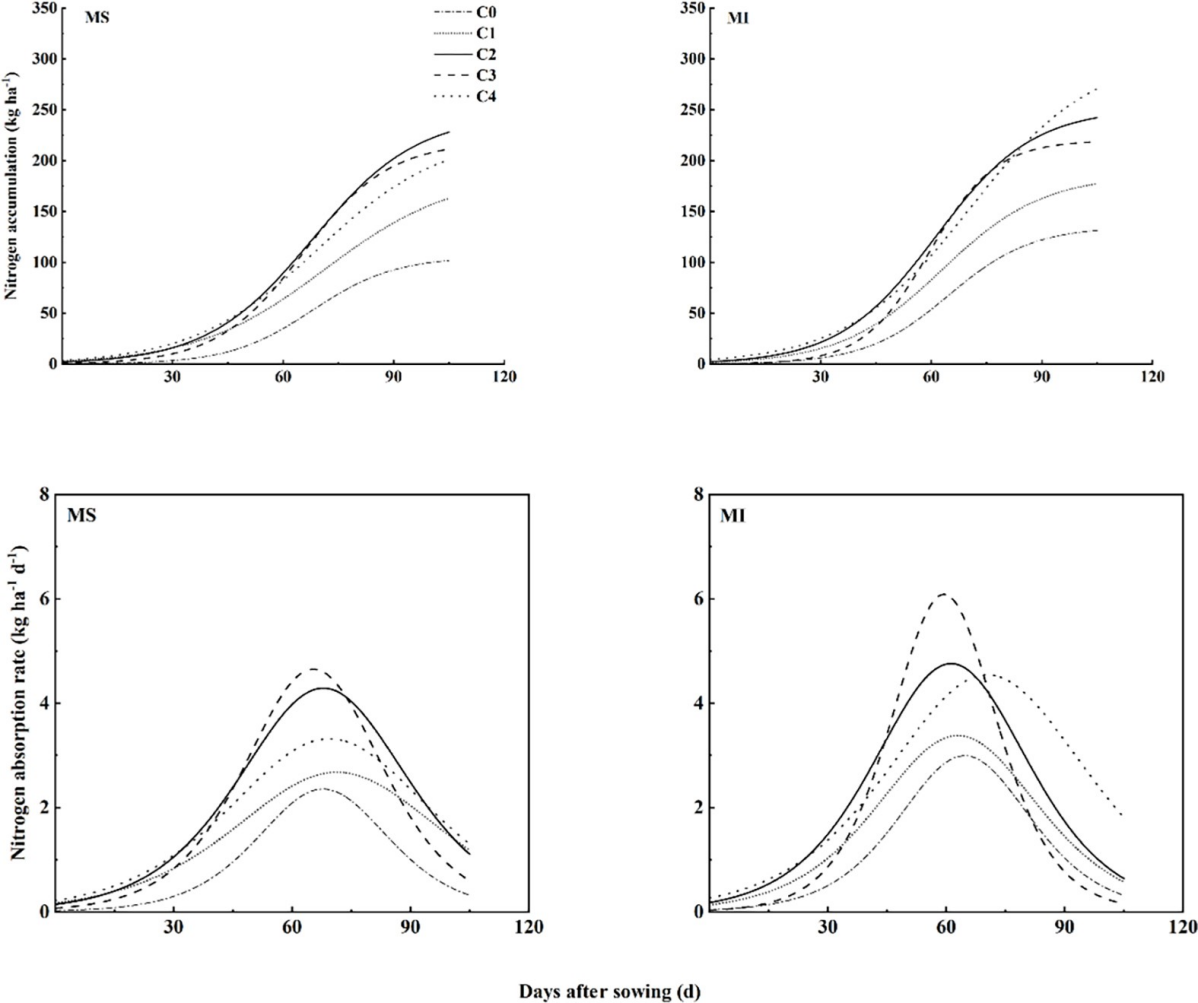
Dynamic curve of nitrogen uptake in spring wheat under different fertilizer rates. Each value is a mean of three replicates and each replicate consisted of five plants (n = 15). MS: spring wheat sole cropping, MI: spring wheat and pea intercropping: C0, C1, C2, C3 and C4 represent 480, 540, 600, and 660 kg ha^-1^ fertilization rate, respectively.

The logistic model simulated the dynamics of aboveground nitrogen accumulation well as indicated by the adjusted R^2^ value which ranges from 0.9614 to 0.9974 (Table 5). Fertilization treatment had extremely significant effects on N (maximum N uptake), v (initial N uptake), T (time to reach maximum N uptake), and V_max_ (maximum absorption rate). The planting pattern had extremely significant effects on N and v. The interaction of fertilization treatment and planting pattern had extremely significant effects on N, v, T, and V_max_. The maximum N uptake of aboveground was highest at C4 under intercropping while lowest at monocropping without fertilizer application (C0) (Fig 1; Table 5). The intercropping in the presence of C2 and C3 fertilizers rate had taken less time to reach maximum N absorption rates which were 61 and 59 days, respectively. while the highest time was taken under monocropping at C1 (71.8 days) and under intercropping at C4 (70.7 days). This indicated that the highest fertilizer rate (C4) under intercropping increased the aboveground N but increased the time to reach that value as compared to C2 and C3. The V_max_ was highest under intercropping at C3 while lowest under monocropping at C0 and C1.

**Table 5:** Key parameters of aboveground nitrogen uptake in spring wheat.

Treatments			Adjusted-R^2^	N (kg ha^-1^)	v (kg ha^-1^ d^-1^)	T (DAS)	V_max_ (kg ha^-1^ d^-1^)
Planting method		MS	-	197.59 b	0.0715 b	68.59 a	3.88 a
		MI	-	220.10 a	0.0810 a	63.82 b	3.90 a
Fertilizers		C0	-	120.27 e	0.0889 b	66.26 ab	2.50 e
		C1	-	187.33 d	0.0641 d	67.37 ab	4.48 b
		C2	-	248.91 b	0.0727 c	64.64 b	3.12 d
		C3	-	219.29 c	0.0978 a	62.43 b	4.04 c
		C4	-	268.42 a	0.0576 e	70.32 a	5.28 a
Interaction							
MS		C0	0.9614	105.50 i	0.0892 b	67.86 bc	2.35 e
		C1	0.9974	188.69 f	0.0560 f	71.77 a	2.64 de
		C2	0.9886	246.27 c	0.0702 de	67.99 bc	4.32 b
		C3	0.9829	218.68 e	0.0850 bc	65.43 cd	4.65 b
		C4	0.9935	228.79 d	0.0570 ef	69.91 ab	3.26 c
MI		C0	0.9923	135.04 h	0.0885 b	64.66 cd	2.99 cd
		C1	0.9811	185.98 g	0.0722 cd	62.97 de	3.36 c
		C2	0.9966	251.55 b	0.0751 cd	61.29 ef	4.72 b
		C3	0.9715	219.90 e	0.1107 a	59.43 f	6.09 a
		C4	0.9820	308.05 a	0.0582 ef	70.74 ab	4.48 b
p-value	Fertilizer			**	**	**	**
	Planting method			**	**	ns	ns
	Interaction			**	**	**	**

MS: spring wheat sole cropping, MI: spring wheat and pea intercropping: C0, C1, C2, C3 and C4 represent 480, 540, 600, and 660 kg ha^-1^ fertilization rate, respectively. Different lowercase letters in the same column indicate significant differences between treatments. N: maximum nitrogen uptake; v: initial nitrogen absorption rate; T: time to reach the maximum nitrogen absorption rate; V_max_: maximum absorption rate. * and ** indicate significance at p < 0.05 and p<0.01, respectively; ns: not significant.

### 3.6. Phosphorus accumulation dynamics in aboveground parts of spring wheat

**Fig 2:**
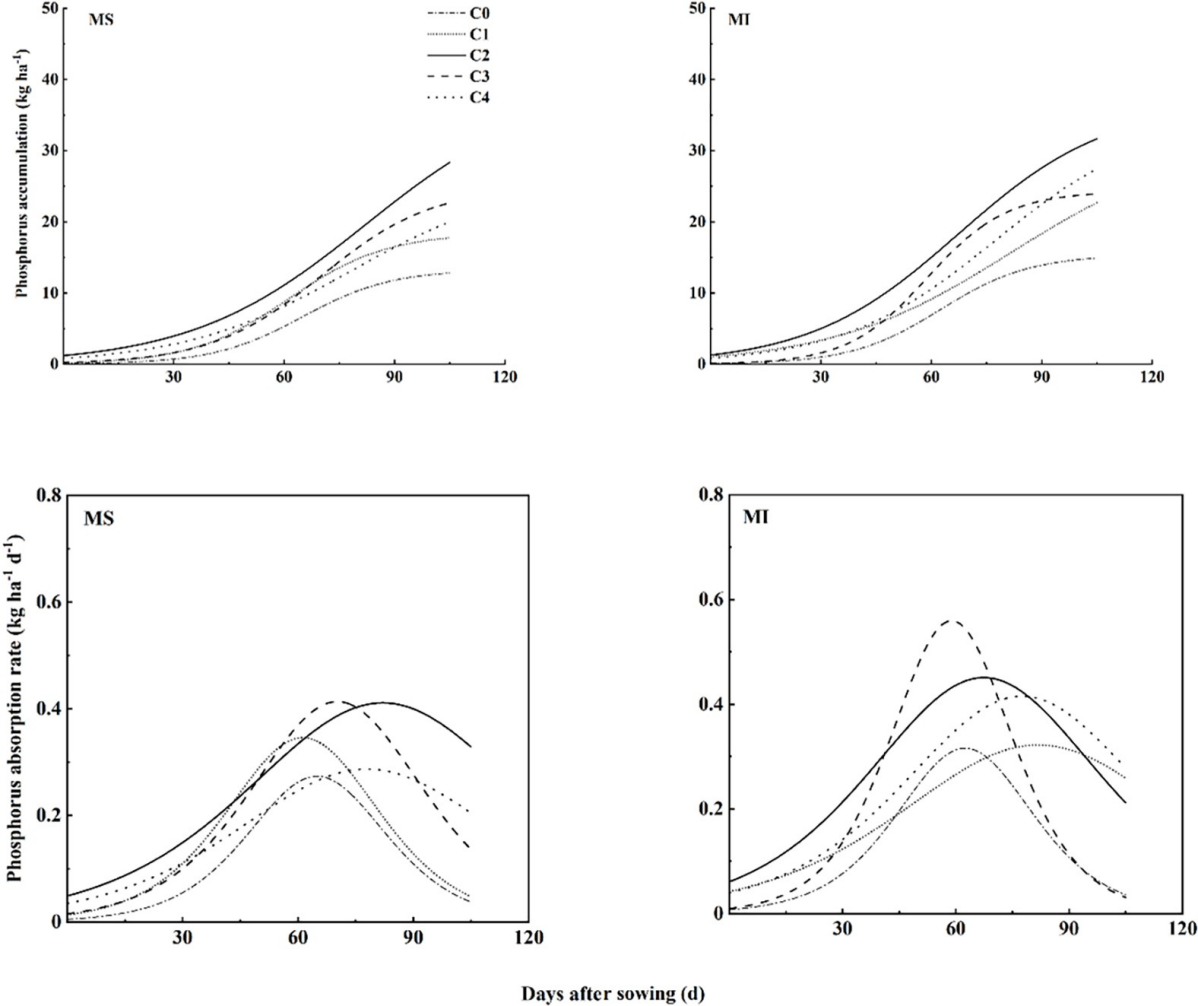
Dynamic curve of phosphorus absorption in spring wheat under different fertilizer rates. Each value is a mean of three replicates and each replicate consisted of five plants (n = 15). MS: spring wheat sole cropping, MI: spring wheat and pea intercropping: C0, C1, C2, C3 and C4 represent 480, 540, 600, and 660 kg ha^-1^ fertilization rate, respectively.

The adjusted R2 value for the logistic simulated model of aboveground phosphorus dynamics ranged from 0.9291 to 0.9788 (Table 6). Planting pattern, fertilization level, and their interaction all had extremely significant effects on P (maximum P uptake), v (initial P uptake), T (time to reach maximum P uptake), and V_max_ (maximum absorption rate). The initial P absorption rate was highest at C3 under monocropping and at C4 under intercropping (Fig 2; Table 6). The minimum P and Vmax were observed at C0 under both cropping methods. The P and Vmax first increased by increasing the fertilizer rate from C0 to C2, reaching a maximum at C2, and then decreased Under both intercropping and monocropping. The C1 under intercropping had taken the highest time (T) to reach the maximum P, followed by C2, while the minimum value of T was at C0 under both cropping systems.

**Table 6:** Key parameters of aboveground phosphorus uptake in spring wheat.

Treatments			Adjusted-R^2^	P (kg ha^-1^)	v (kg ha^-1^ d^-1^)	T (DAS)	V_max_ (kg ha^-1^ d^-1^)
Planting method		MS	-	26.11 b	0.0636 a	77.50 a	0.42 b
		MI	-	29.05 a	0.0626 a	78.93 a	0.46 a
Fertilizers		C0	-	14.57 c	0.0686 ab	70.19 c	0.28 c
		C1	-	30.20 b	0.0518 b	84.57 a	0.57 a
		C2	-	46.70 a	0.0527 b	88.27 a	0.30 c
		C3	-	28.53 b	0.0734 a	76.67 b	0.53 b
		C4	-	26.73 b	0.0661 ab	75.66 b	0.53 b
Interaction							
MS		C0	0.9291	13.60 f	0.0691 b	70.17 e	0.24 f
		C1	0.9566	21.54 e	0.0589 c	73.57 de	0.32 e
		C2	0.9746	44.14 b	0.0550 d	88.58 b	0.61 a
		C3	0.9567	27.57 d	0.0784 a	77.89 c	0.54 b
		C4	0.9584	23.72 e	0.0564 cd	77.26 cd	0.34 e
MI		C0	0.9469	15.54 f	0.0680 b	70.22 e	0.26 f
		C1	0.9788	38.87 c	0.0446 f	95.57 a	0.43 d
		C2	0.9714	49.26 a	0.0503 e	87.95 b	0.62 a
		C3	0.9671	29.50 d	0.0683 b	75.45 cd	0.50 c
		C4	0.9459	29.73 d	0.0758 a	74.06 cde	0.56 b
p-value	Fertilizer			**	**	**	**
	Planting method			**	**	**	**
	Interaction			**	**	**	**

MS: spring wheat sole cropping, MI: spring wheat and pea intercropping: C0, C1, C2, C3 and C4 represent 480, 540, 600, and 660 kg ha^-1^ fertilization rate, respectively. Different lowercase letters in the same column indicate significant differences between treatments. P: maximum phosphorus content accumulation; v: initial phosphorus absorption rate; T: time to reach the maximum phosphorus absorption rate; V_max_: maximum phosphorus absorption rate

* and ** indicate significance at p < 0.05 and p<0.01, respectively; ns: not significant.

## 4. Discussion

Intercropping, as a practice where multiple species share the same land, can intensify intra-species competition for nutrients, thereby promoting more efficient nutrient utilization during crop growth and development. This strategy, when implemented judiciously, can enhance crop productivity and resource use efficiency, thus contributing to sustainable agricultural development [23]. Legume-cereal intercropping stands as a prime example of multifunctional agroecosystems [24], known for its ability to boost nutrient contents in crops, improve yields, and establish high-yielding ecosystems [25].

Consistent with these findings, our study demonstrates that intercropping of spring wheat with peas resulted in significant increases in spike number, grains per spike, 1000-grain weight, and grain yield. Notably, we observed that spring wheat maintained high yields even with a 10% reduction in fertilizer application (C2), suggesting that reduced fertilization could adequately meet the nutrient requirements of intercropped spring wheat, consequently enhancing grain yield, which aligns with previous research [26].

However, our investigation also sheds light on the complex relationship between fertilizer application rates and fertilizer use efficiency. Contrary to conventional expectations, we found that fertilizer use efficiency did not exhibit a linear relationship with application rates; instead, it showed a decrease with increasing fertilization. This phenomenon could be attributed to the point at which soil nutrient inputs surpass crop demand, leading to a gradual increase in soil fertility indices and diminishing crop yield responses, ultimately impacting fertilizer use efficiency negatively [27]. Therefore, optimizing fertilization rates is crucial not only to ensure adequate nutrient supply to crops but also to enhance fertilizer use efficiency and mitigate environmental issues associated with excessive fertilizer application.

As our study delved into the dynamics of nutrient accumulation in spring wheat, we employed the Logistic model to simulate aboveground nitrogen and phosphorus accumulation patterns effectively. The observed S-shaped curve for nutrient accumulation over time, along with bell-shaped uptake rate curves, is consistent with previous findings [7]. Intercropping of legumes with cereals has been shown to promote nitrogen and phosphorus uptake in cereals, owing to the complementary nutrient acquisition strategies of these crops [28, 29]. Our study corroborates these findings, demonstrating significantly higher values for key nitrogen and phosphorus uptake parameters under intercropping compared to monoculture. This enhanced nutrient uptake and accumulation under intercropping conditions could be attributed to legume-root-mediated nitrogen transfer, which facilitates nitrogen uptake and accumulation in spring wheat [30, 31]. Furthermore, legume nitrogen fixation plays a crucial role in improving cereal nitrogen nutrition, thereby enhancing cereal phosphorus uptake and promoting spring wheat phosphorus uptake and accumulation [28, 32].

Our investigation also underscores the significant impact of fertilizer application on spring wheat nutrient accumulation. We found that fertilization significantly increased maximum nitrogen accumulation, maximum nitrogen uptake rate, and maximum phosphorus accumulation, highlighting the critical role of fertilization in meeting crop nutrient demands during growth and development [7, 33]. Notably, appropriate reductions in nitrogen application were found to enhance nitrogen use efficiency [34–36], while reduced fertilizer application significantly improved wheat phosphorus uptake efficiency in our study. This suggests that optimizing fertilizer rates can maintain high nutrient accumulation while improving nutrient uptake efficiency, thus mitigating environmental burdens associated with excessive fertilizer use.

In summary, our findings demonstrate that intercropping practices, in conjunction with optimized fertilization strategies, can significantly enhance nutrient uptake and utilization efficiency in spring wheat-pea intercropping systems. Synthesizing the effects of different planting patterns and fertilization treatments, we observed a close relationship between nutrient uptake and yield, highlighting the potential of intercropping to promote sustainable crop production.

## 5. Conclusion

Intercropping along with appropriate fertilization increased spring wheat yield and promoted nitrogen and phosphorus accumulation. The optimum fertilizer dose for maximum yield was 540 kg h^-1^ which was 10% less than the conventional rate. Increasing the fertilizer rate not only reduced spring wheat yield and yield components but also slowed down nitrogen and phosphorus accumulation. Therefore, nutrient inputs should be balanced in the intercropping system to maximize intercropping advantages while avoiding fertilizer excess issues. Reasonable fertilizer management is key to optimizing nutrient accumulation, yield, and economic benefits.

## Supporting information

S1 Fig The average monthly maximum temperature, average monthly minimum temperature and precipitation in the field test sites during the whole growing period from 2022 to 2023. (DOCX)
