# Supplementary material for: Leveraging logistic models to enhance nutrient dynamics modeling in intercropped spring wheat with varied nitrogen and phosphorus fertilization strategies
Source: PLoS One. 2024 Dec 5;19(12):e0314264. doi: 10.1371/journal.pone.0314264 (PMC11620655; doi:10.1371/journal.pone.0314264)
Supplement: S1 Fig — (DOCX) [file pone.0314264.s001.docx]

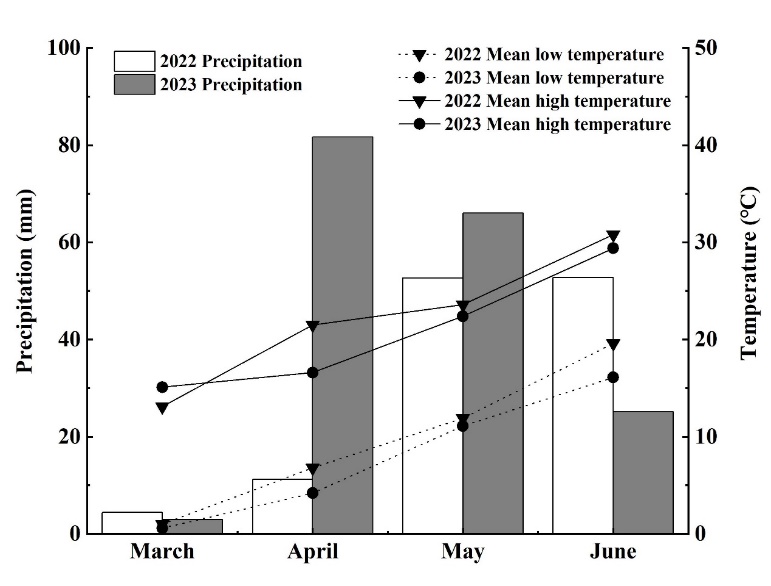


**Fig. S1** The average monthly maximum temperature, average monthly minimum temperature and precipitation in the field test sites during the whole growing period from 2022 to 2023.
